# Population subdivision of the surf clam *Mactra chinensis* in the East China Sea: Changjiang River outflow is not the sole driver

**DOI:** 10.7717/peerj.1240

**Published:** 2015-09-29

**Authors:** Gang Ni, Qi Li, Lehai Ni, Lingfeng Kong, Hong Yu

**Affiliations:** 1 The Key Laboratory of Mariculture, Ministry of Education, Ocean University of China, Qingdao, China; 2 Shandong Fisheries Technical Extension Station, Jinan, China

**Keywords:** Population subdivision, Biogeographic boundary, Intertidal invertebrates, Northwestern Pacific, Marine phylogeography

## Abstract

The northwestern Pacific, characterized by unique tectonic and hydrological settings, has greatly intrigued marine phylogeographers. However, current studies mostly focus on the influence of Pleistocene isolation of sea basins in population structure of species in the region, leaving the contribution of other factors (such as freshwater outflow and environmental gradients) largely unexploited. Here we shed light on the question by investigating phylogeography of the surf clam *Mactra chinensis* in the East China Sea (ECS). Genetic information was acquired from 501 specimens collected from its main distribution in the region, represented by mitochondrial cytochrome oxidase I (COI) and nine polymorphic microsatellite loci. A shallow and star-like phylogeny was revealed for all COI haplotypes, indicating the origin of populations from a single refugium. Although no divergent lineages existed, population subdivision was detected in both data sets. The most striking pattern was the significant differentiation between populations north and south of a biogeographic boundary—the Changjiang Estuary, suggesting a barrier effect of the freshwater outflow to gene flow. For the northern group, substructure was revealed by COI result as one southernmost population was significant different from other ones. Clear latitude gradations in allele frequencies were revealed by microsatellite analyses, likely influenced by environmental gradient factors such as temperature. Our results demonstrate that genetic subdivision can arise for populations within the ECS despite they have a single origin, and multiple mechanisms including Changjiang River outflow, environmental gradient factors and life-history traits may act in combination in the process.

## Introduction

Pinpointing mechanisms facilitating marine population subdivision and speciation has long intrigued biogeographers (e.g., Mayr, 1954; Palumbi, 1992; Miglietta, Faucci & Santini, 2011). For the vast region of the northwestern (NW) Pacific, our understanding of its origin of population subdivision however is seriously biased. Current information is mainly obtained from physical isolation of populations in sea basins (e.g., Liu et al., 2007b; Kong & Li, 2009; Xu et al., 2009; Liu et al., 2011; Ni et al., 2012), leaving the contribution of other factors (such as freshwater outflow, habitat discontinuity and environmental gradients) largely unexplored.

**Figure 1:**
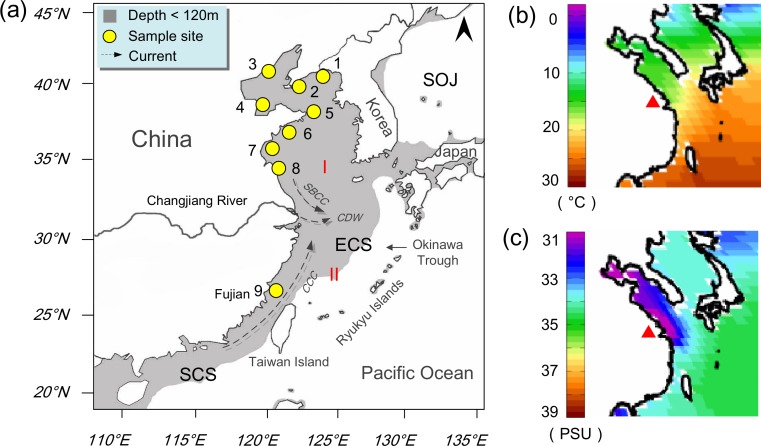
Sampling map. Study area in the northwestern Pacific showing sample sites of *Mactra chinensis* and environmental factors. (A) Sampling locations and the coastal current system in summer. Populations are marked with numbers as shown in Table 1. *SBCC*, Subei Coastal Current; *CDW*, Changjiang Diluted Water; *CCC*, China Coastal Current; SOJ, Sea of Japan; ECS, East China Sea; SCS, South China Sea. I, Far East Subregion of North Pacific Region; II, Sino-Japanese Subregion of Indo-West Pacific Region (Zhang et al., 1963). (B) Annual mean sea surface temperature (in degrees Celsius, °C) in the study area, modified from the World Ocean Atlas 2001 (Conkright et al., 2001); (http://www.nodc.noaa.gov/). The red triangle indicates the location of the Changjiang Estuary. (C) Annual mean sea surface salinity (in Practical Salinity Units, PSU) in the study area, modified from the World Ocean Atlas 2001.

The East China Sea (ECS) with the Bohai Gulf and the Yellow Sea (hereafter considered as a single sea with no barriers among them, Fig. 1), as one of the largest marginal seas in the world (Xu & Oda, 1999), represents a useful system to examine whether and how population subdivision can arise within a peripheral ecosystem. It is characterized by a shallow and extensive continental shelf with a total area of 850,000 km^2^, which was likely subjected to amplified sea-level-induced environmental changes through the Pleistocene (Wang et al., 1997): when glaciers advanced, the sea level of the ECS declined about 130–150 m than present, resulting in exposure of the entire shelf (Xie, Jian & Zhao, 1995), and the sea retreated to an elongate and curved back arc basin—the Okinawa Trough with an area of <1/3 of its present size (Wang, 1999); when sea levels rose during inter-glaciations, the coastline migrated landwards from the western border of the basin to the modern ECS coast, causing the inundation of the ECS shelf (Xu & Oda, 1999). Such drastic sea level fluctuations could have dramatically influenced the genetic pattern of species occurring in the ECS, especially for intertidal species having experienced a direct loss of habitat (Ni et al., 2012). They would have been forced into the refugium during the contraction of the ECS, and migrated out following the flooding, rapidly re-populating the continental shelf (McManus, 1985). Newly established populations after the last glacial maximum (LGM) were expected to carry similar genetic signals as they might be derived from a panmictic ancestral population in the basin (Liu et al., 2011). The pattern would be reinforced by contemporary oceanographic conditions as several coastal current systems in the region may facilitate connectivity among populations (Su & Yuan, 2005).

Meanwhile, the ECS spans a biogeographic boundary for molluscan fauna—the Changjiang Estuary (31°N), separating (I) Far East Subregion of North Pacific Region from (II) Sino-Japanese Subregion of Indo-West Pacific Region (Fig. 1; Zhang et al., 1963). Studies have suggested province boundaries constraining species’ spatial ranges often generate intraspecific genetic structure for those species whose ranges span them (Brante, Fernández & Viard, 2012). As the third largest river in the world, the Changjiang (also named the Yangtze) River enters the ECS with an average annual discharge of over 900 billion m^3^. The huge freshwater outflow collides with the southward Subei Coastal Current and northward East China Sea Coastal Current, resulting in strong water property gradients (e.g., temperature and salinity) (Fig. 1). Marked associated changes include coastal topography, sediment types and nutrient conditions in the vicinity of the estuary (Wang, Wang & Zhan, 2003; Liu et al., 2010), which may act in combination as a barrier for gene flow. Indeed, there have been several studies focusing on the barrier effect of the freshwater on the intraspecific level (e.g., Kong, Li & Qiu, 2007; Dong et al., 2012; Ni et al., 2012). However, the observed divergence in the studies above could not be simply attributed to the freshwater barrier because it might originate from the isolation of sea basins followed by recent colonization. Furthermore, the genetic markers they applied were mainly mitochondrial markers, which had limited power to infer population subdivision at recent time scales. Microsatellites, characterized by extremely high level of polymorphism, could provide higher resolution to detect population differentiation (Pedreschi et al., 2014).

In this study, we attempt to shed light on evolutionary process within the ECS, using the surf clam *Mactra chinensis* Philippi 1846 (Mollusca, Bivalvia, Mactridae) as a model organism, employing genetic data from mitochondrial cytochrome oxidase I (COI) and polymorphic microsatellite markers. *Mactra chinensis* is a temperate bivalve with distribution in the northwestern Pacific, and only occurs naturally in the ECS along Chinese coastline (Xu & Zhang, 2008). This distributional pattern suggests that the current ECS populations were most likely formed via *in situ* glacial survival in the ECS basin. In the ECS, *M*. *chinensis* appears mostly north of the Changjiang Estuary, burrowed in fine sand bank from low-tide level to shallow waters <60 m, but with an isolated population in the southern Fujian Province (Xu, 1997). The clam is characterized by high fecundity (>10^6^ eggs per female) and a pelagic larval duration (PLD) up to 10 days (Wang et al., 1984), suggesting dispersal potential via coastal currents. In experimental conditions, the embryo can develop at salinity of 18–30 PSU and temperature of 20–28 °C (Liu et al., 2007a). The larvae have a survival rate of nearly 100% during the pelagic stage from day 0 to day 10 at about 23 °C. Higher temperature (>30 °C) can cause high mortality. When the salinity is <15 PSU, the development of larvae will be significantly refrained. These abiotic and biotic factors may have complex interactions on the population structure of *M. chinensis*, which need to be carefully disentangled. Here we will first examine whether the current ECS populations have a single origin, and then test whether there is genetic subdivision among the populations. We will discuss the results in the context of potential influence of diverse factors.

## Materials and Methods

### Sample collection and DNA extraction

Sampled sites were chosen to cover the main natural range of *M. chinensis* in the ECS according to the description of Xu (1997). As it is not endangered or protected species, no specific permit is required for collection. Finally, a total of 501 specimens of nine natural populations were sampled from public access areas between May 2006 and September 2010 (Table 1). We failed to collect any populations from Lianyungang (LY) to Pingtan (PT) with a straight-line distance of approximately 1,000 km during our several years’ fieldwork, which was mainly due to unsuitable habitat types (mud substrate) around the estuary. The adductor muscle was excised from each individual and preserved in 100% ethanol immediately until DNA preparation. Genomic DNA was extracted from approximate 50 mg muscle tissue using a standard phenol-chloroform extraction method as described by Li, Park & Kijima (2002).

**Table 1:** Sampling details and diversity indices (with standard deviation) for the nine populations of Mactra chinensis.

Sample site (Abbr.)	Latitude, longitude	Mitochondrial diversity				Microsatellite diversity			
		*N*	*n*	*h*	*π*	*N*	*A_R_*	*H_E_*	*H_O_*
1. Dandong (DD)	39°50′N, 124°10′E	23	6	0.632 (0.090)	0.00230 (0.00161)	42	16.56 (3.60)	0.87 (0.07)	0.56 (0.09)
2. Zhuanghe (ZH)	39°38′N, 122°59′E	16	5	0.533 (0.142)	0.00193 (0.00145)	60	17.07 (3.42)	0.89 (0.05)	0.67 (0.07)
3. Qinhuangdao (QH)	39°54′N, 119°36′E	18	5	0.549 (0.127)	0.00148 (0.00119)	50	17.38 (4.24)	0.88 (0.07)	0.58 (0.11)
4. Penglai (PL)	37°50′N, 120°44′E	21	9	0.757 (0.088)	0.00224 (0.00160)	60	17.63 (4.08)	0.90 (0.04)	0.54 (0.12)
5. Wendeng (WD)	36°54′N, 122°03′E	14	9	0.879 (0.079)	0.00373 (0.00243)	55	16.49 (3.59)	0.88 (0.08)	0.56 (0.11)
6. Haiyang (HY)	36°34′N, 121°01′E	15	10	0.942 (0.040)	0.00305 (0.00206)	54	17.11 (3.25)	0.90 (0.04)	0.53 (0.17)
7. Rizhao (RZ)	35°06′N, 119°23′E	20	9	0.790 (0.086)	0.00250 (0.00174)	60	16.59 (3.81)	0.87 (0.08)	0.48 (0.11)
8. Lianyungang (LY)	34°34′N, 119°41′E	19	10	0.784 (0.098)	0.00350 (0.00226)	60	17.17 (3.35)	0.88 (0.06)	0.63 (0.07)
9. Pingtan (PT)	25°30′N, 119°54′E	20	9	0.705 (0.114)	0.00390 (0.00245)	60	14.27 (5.11)	0.81 (0.09)	0.57 (0.09)

**Notes.**

*N* number of individuals analysed *n* number of haplotypes *h* haplotype diversity *π* nuclear diversity *A_R_* mean allele richness *H_E_* expected heterozygosity *H_O_* observed heterozygosity Abbr site abbreviation

### Mitochondrial DNA sequencing and analysis

The mitochondrial COI gene was amplified for a subset of individuals (14 to 23 specimens) in each population with the primers LCO-1490 and HCO-2198 (Folmer et al., 1994). Each polymerase chain reaction (PCR) was performed in 50-µL volumes containing 2 U *Taq* DNA polymerase (Takara, Otsu, Shiga, Japan), 50–100 ng of genomic DNA, 0.25 µM of each primer, 0.2 mM dNTP mix, 2 mM MgCl_2_ and 5 µL 10× PCR buffer. PCR was carried out on a GeneAmp^®^ 9700 PCR System (Applied Biosystems, Carlsbad, California, USA) based on the conditions in Ni et al. (2012). Amplification products were confirmed by 1.5% TBE agarose gel electrophoresis and then purified using EZ Spin Column PCR Product Purification Kit (Sangon, Shanghai, China) following described protocol. The cleaned product was prepared for sequencing using the BigDye Terminator Cycle Sequence Kit (ver. 3.1; Applied Biosystems) and finally analysed on an ABI PRISM 3730 automatic sequencer. Sequences were assembled and aligned using the DNAstar software suite (DNASTAR, Madison, Wisconsin, USA). Haplotypes were defined using the DnaSP 5 (Librado & Rozas, 2009), and their relationships were inferred using a maximum parsimony network in the TCS 1.21 package (Clement, Posada & Crandall, 2000). The best-fit model of sequence evolution was determined with jModelTest 2 (Darriba et al., 2012). GTR + I model was selected under the Akaike information criterion and used in subsequent analysis. Molecular diversity indices such as number of haplotypes (*n*), haplotype diversity (*h*) and nucleotide diversity (*π*) were calculated for each population in the ARLEQUIN 3.5 (Excoffier & Lischer, 2010).

A spatial analysis of molecular variance (SAMOVA) was used to define the best population grouping strategy based on *F*_CT_ values (number of groups *K*: 2 to 8) in SAMOVA 1.0 (Dupanloup, Schneider & Excoffier, 2002). A hierarchical analysis of molecular variance (AMOVA; Excoffier, Smouse & Quattro, 1992) was conducted with 10,000 permutations in ARLEQUIN 3.5 to estimate the partitioning of genetic variation. As the GTR model was not available in ARLEQUIN, the closest model Tamura & Nei (1993) was used. Pairwise Φ_ST_ was also calculated under this model with 1,000 random replicates followed by a standard Bonferroni correction (Rice, 1989). The mantel test (1,000 randomizations) for isolation by distance (IBD) was performed between genetic similarity (*F*_ST_/(1 − *F*_ST_)) (Slatkin, 1995) and Euclidean geographical distances using IBDWS version 3.23 (Jensen, Bohonak & Kelley, 2005).

Historical demography of each population was investigated using Tajima’ *D* (Tajima, 1989) and Fu’s *F_S_* neutrality tests (Fu, 1997) as implemented in ARLEQUIN 3.5 with 10,000 bootstrap replicates. Once a test yielded a value that was significantly different from zero, mismatch distribution was performed to further characterize the expansion. The sum-of-squared-differences (SSD) statistic was used to test the goodness-of-fit between the observed mismatch distribution and that expected under a sudden expansion model (10,000 replicates).

The coalescent approach implemented in IMa (Hey & Nielsen, 2007) was used to parameterize gene flow and divergence time among three groups (namely G1, G2 and G3, see results). Divergence time (*t*) was individually estimated for two group pairs G1:G2 and G1:G3 because G2 and G3 seemed to derive from G1 separately. Initial runs were analysed to determine the appropriate upper bounds for the migration (*m*) and divergence time (*t*). Twenty heated Markov chains were run with a burn-in period of 10 million steps, and all runs were consisted of 100 million steps (recording every 1,000 steps). Heating parameters (*g*1 = 0.8 and *g*2 = 0.9) were used to provide good mixing of the Markov chains. Each procedure was repeated at least for three times with different random seeds. The analyses were considered to converge upon a stationary distribution if the independent runs reported similar posterior distributions (Hey, 2005) and the ESS for each run was >200 (Won & Hey, 2005). The mutation-scaled parameter *t* can be converted into the real time (*T*) based on following formula: *T* = *t* × *g*/*u* (*g*, generation time; *u*, mutation rate per locus per year) (Hey & Nielsen, 2004). For *M. chinensis*, the generation time was 1 year (Wang et al., 1984). However there was neither an accurate mutation rate nor a clear fossil record available for the species. Former molluscan studies using mutation rates estimated from deep splits of interspecific phylogeny were recently questioned because accelerated molecular rate estimates were suggested in short evolutionary timescales, known as the “time dependency molecular rates” hypothesis (Ho et al., 2005). Under the hypothesis, mutation rate can be an order of magnitude faster than that based on a phylogenetic calibration (Grant et al., 2012). So we adopted here a tenfold faster mutation rate of 12% myr^−1^ than the upper boundary (1.2% myr^−1^) used in former studies (e.g., Marko et al., 2010; Liu et al., 2011) to shed light on a recent demographic scenario.

### Microsatellite genotyping and analysis

Microsatellite data were analysed to validate the population structure revealed by mitochondrial COI. In our previous study, we had genotyped eight populations (DD, ZH, QH, PL, WD, HY, RZ and LY) with nine polymorphic microsatellites (Ni, Li & Kong, 2011). Here we screened 60 individuals in PT population with the same microsatellite loci. A detailed methodology of PCR and genotyping conditions can be found in Ni, Li & Kong (2011).

The expected heterozygosity (*H_E_*) and observed heterozygosity (*H_O_*) were calculated for each population using the program MICROSATELLITE ANALYSER (MSA; Dieringer & Schlotterer, 2003), and the mean allele richness (*A_R_*) was calculated with FSTAT version 2.9.3 (Goudet, 2001). Weir & Cockerham’s (1984) *F*_ST_ (*θ*) was calculated in the program MSA (Dieringer & Schlotterer, 2003). Significance of values was tested using 1,000 permutations with the Bonferroni correction (Rice, 1989). The mantel test (1,000 randomizations) for IBD was performed using the combined data set of 501 individuals in IBDWS.

We used two different kinds of analyses to assess the population structure. First, the Cavallis-Sforza & Edwards (1967) chord distance *D_C_* was calculated and an unrooted neighbor-joining (NJ) tree was generated using the software POPULATIONS v1.2.31 (Langella, 2011). Supports for nodes were assessed by bootstrapping with 1,000 replicates. Second, population structure was inferred with a Bayesian algorithm as implemented in STRUCTURE v.2.3 (Pritchard, Stephens & Donnelly, 2000). Tested *K* ranged from 1 to 12 (sampled populations plus three). For each value, 20 replicates were run using the admixture model, correlated allele frequencies and the prior population information with a burn-in period of 10,000, followed by 100,000 steps. The most appropriate value of *K* was determined by the statistic Δ*K* introduced by Evanno, Regnaut & Goudet (2005) using Structure Harvester v0.6.92 (Earl & von Holdt, 2012). We averaged all 20 replicates for the best *K* by Greedy method implemented in CLUMPP (Jakobsson & Rosenberg, 2007), and finally visualized the results with DISTRUCT (Rosenberg, 2004). AMOVA analysis was conducted with 10,000 replicates in ARLEQUIN to check for hierarchical structure of variability.

## Results

### Mitochondrial COI analyses

**Figure 2:**
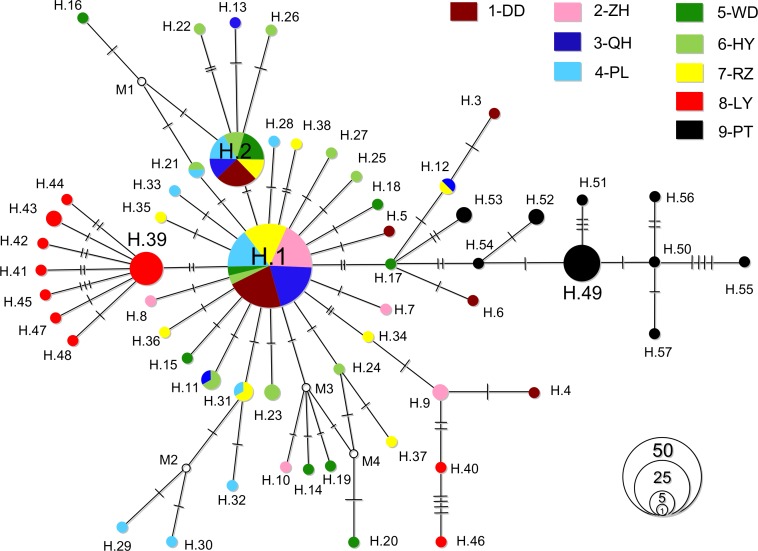
Haplotype network. Haplotype relationships of *M. chinensis*. Each circle represents a single haplotype sized in proportion to its frequency. Bars indicate mutation steps between haplotypes. Small empty circles (M1-4) represent missing haplotypes.

An alignment of 627 bp COI gene was analysed for the 166 individuals, with 59 variable sties yielding a total of 57 unique haplotypes (deposited in GenBank with accession numbers KC205870–KC205926). The number of haplotypes per population ranged from 5 to 10 with an average of 8. Haplotype diversity ranged from 0.533 in ZH to 0.942 in HY, and nuclear diversity ranged from 0.00148 in QH to 0.00390 in PT (Table 1). The parsimony network roughly showed a ‘star-like’ topology with significant geographic patterns (Fig. 2). The central haplotype (H.1) was the most common one with 59 copies (accounting for 35.5%), and appearing in seven of nine populations, it was the most likely ancestral haplotype according to Posada & Crandall (2001). H.1 was separated from the other haplotypes by at most 10 mutation steps. The second haplotype H.2, appearing in six populations, was also abundant (24 copies, 14.5%). Haplotypes H.39-48, however, were spatially restricted to LY with no sharing with other populations noticed. The situation was also observed for PT with all nine haplotypes H.49-57 private (Fig. 2).

**Figure 3:**
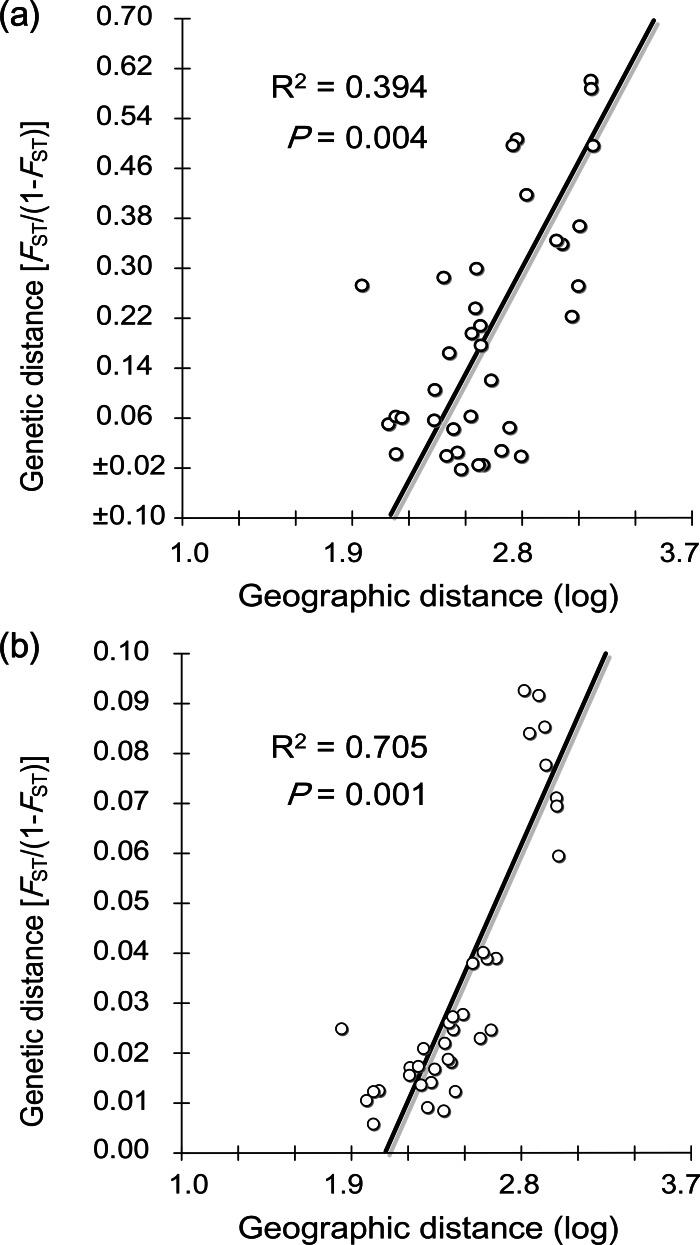
Isolation by distance analyses for *M. chinensis* populations. (A) Relationship between geographic (log-transformed) and COI genetic distances. (B) Relationship between geographic (log-transformed) and microsatellite genetic distances.

For SAMOVA analysis, the grouping for which the *F*_CT_ was highest was chosen as the optimal grouping method. Here the highest *F*_CT_ appeared at three groups ((DD, ZH, QH, PL, WD, HY, RZ); (LY); (PT), referred to hereafter as G1, G2 and G3 correspondingly). Based on this strategy, hierarchical analyses of AMOVA revealed a significant level of geographic structuring among groups (Φ_CT_ = 0.6574, *P* = 0.0262; Table 2). Genetic differences among groups explained 65.74% of the total variation, followed by variation within populations of 33.83% (Φ_ST_ = 0.6617). Only 0.43% of the variation was attributed to variation among populations within groups (Φ_SC_ = 0.0124). These results were corroborated with the pairwise Φ_ST_ values: genetic structure was indicated as 17 comparisons were significant after Bonferroni corrections (Table 3). LY and PT showed remarkably differentiation from each other and all other sites with rather large Φ_ST_ (0.484–0.726 and 0.616–0.726, respectively). The IBD analysis suggested significantly positive correlation between genetic and geographic distance matrices (*R*^2^ = 0.394, *P* = 0.004; Fig. 3A).

**Table 2:** AMOVA analyses. Results from analysis of molecular variance (AMOVA) of population structure in Mactra chinensis.

Marker	Grouping	Source of variation	*df*	Statistics	% of variation	*P* value
Mitochondrial COI	(DD, ZH, QH, PL,WD, HY, RZ); (LY); (PT)	Among groups	2	Φ_CT_ = 0.6574	65.74	0.0262^*^
		Among populations within groups	6	Φ_SC_ = 0.0124	0.43	0.0257^*^
		Within populations	157	Φ_ST_ = 0.6617	33.83	<0.0001^*^
Microsatellites	(DD, ZH, QH, PL,WD, HY, RZ, LY); (PT)	Among groups	1	*F*_CT_ = 0.0521	5.21	<0.0001^*^
		Among populations within groups	7	*F*_SC_ = 0.0234	2.22	<0.0001^*^
		Within populations	993	*F*_ST_ = 0.0743	92.57	<0.0001^*^

**Notes.**

* significant at *P* < 0.05.

**Table 3:** Population comparison. Pairwise Φ_ST_ (COI, below diagonal) and *F*_ST_ (*θ*) values (microsatellites, above diagonal) between the *M. chinensis* populations.

Sites	DD	ZH	QH	PL	WD	HY	RZ	LY	PT
DD	—	0.010^*^	0.012^*^	0.018^*^	0.016^*^	0.027^*^	0.024^*^	0.037^*^	0.056^*^
ZH	0.041	—	0.014^*^	0.009^*^	0.017^*^	0.018^*^	0.022^*^	0.037^*^	0.067^*^
QH	−0.031	0.058	—	0.013^*^	0.021^*^	0.024^*^	0.036^*^	0.038^*^	0.066^*^
PL	0.021	0.061	0.012	—	0.005	0.012^*^	0.016^*^	0.025^*^	0.073^*^
WD	0.016	0.104^*^	0.026	0.051	—	0.012^*^	0.008^*^	0.026^*^	0.079^*^
HY	0.008	0.084^*^	−0.003	0.010	−0.001	—	0.015^*^	0.020^*^	0.085^*^
RZ	−0.013	0.013	−0.020	−0.008	0.039	0.011	—	0.024^*^	0.078^*^
LY	0.529^*^	0.531^*^	0.559^*^	0.532^*^	0.484^*^	0.498^*^	0.511^*^	—	0.085^*^
PT	0.680^*^	0.686^*^	0.706^*^	0.697^*^	0.616^*^	0.667^*^	0.674^*^	0.726^*^	—

**Notes.**

* Indicates significant difference after Bonferroni correction (*P* < 0.05/36).

Only two populations ZH and PT showed no signal of historical expansion in both Tajima’ *D* and Fu’ *F_S_* tests (*P* > 0.05, Table 4). For the rest, at least one of the tests yielded significantly negative value, indicating each population had experienced a demographical expansion under the neutral model (Rogers & Harpending, 1992). The subsequent goodness-of-fit tests for them also supported the null hypothesis of sudden expansion model with nonsignificant values for SSD (all *P* > 0.05, Table 4).

**Table 4:** Demongraphy analyses. Estimates of neutral tests (Tajima’ *D* and Fu’ *F_S_*) for each population and the mismatch distribution parameter SSD.

Population	Tajima’ *D*		Fu’ *F_S_*		Goodness-of-fit test	
	*D*	*P*	*F_S_*	*P*	SSD	*P* (Sim. Ssd ≥ Obs. Ssd)
1. DD	−1.7792	0.0187	−0.9806	0.2718	0.0383	0.05700
2. ZH	−1.5192	0.0556	−0.9669	0.2009	—	—
3. QH	−1.5531	0.0463	−1.4663	0.0975	0.0043	0.7510
4. PL	−1.4943	0.0572	−4.7706	0.0006	0.0083	0.3840
5. WD	−1.5354	0.0511	−4.1843	0.0053	0.0115	0.4300
6. HY	−1.4488	0.0695	−6.4180	0.0000	0.0438	0.0740
7. RZ	−2.0852	0.0064	−4.4322	0.0016	0.0011	0.9000
8. LY	−2.2006	0.0024	−4.4153	0.0058	0.0024	0.9540
9. PT	−1.4197	0.0660	−2.5527	0.0721	—	—

**Figure 4:**
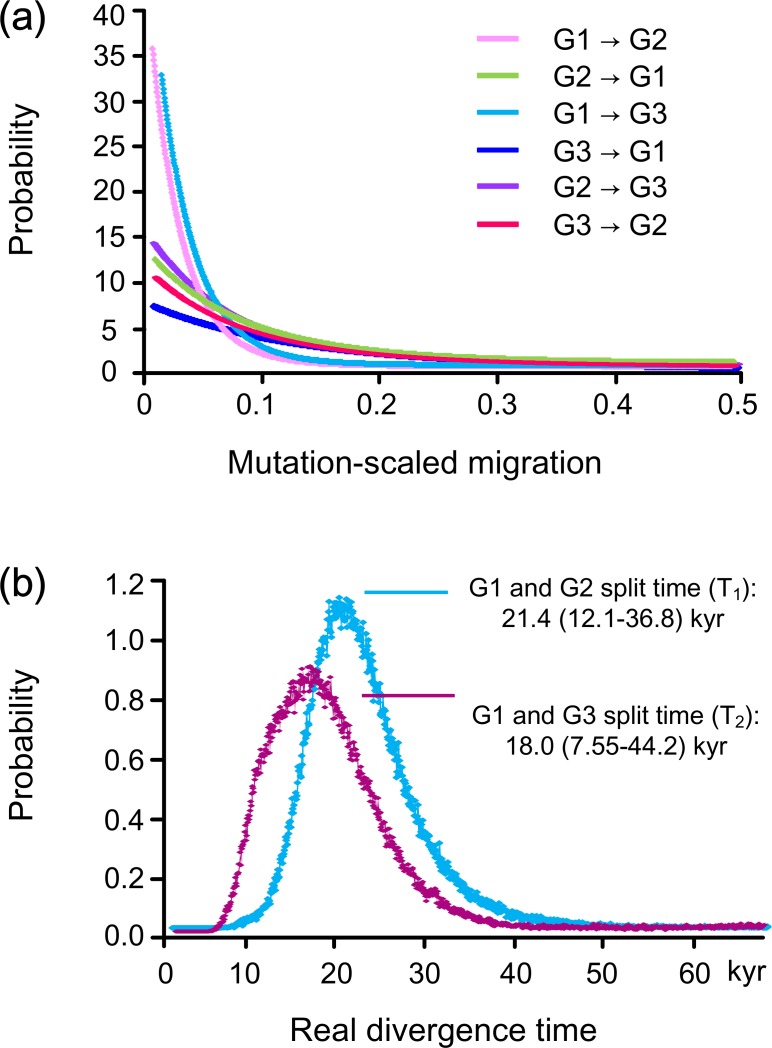
IMa analyses. (A) Gene flow and (B) divergence time among the three groups (G1, G2 and G3) estimated using coalescent approach IMa.

Independent runs of IMa2 converged on similar marginal posterior probability distributions. Plots of the probability for the migration parameters of pairwise groups were uniformly unambiguous and very narrow: each of them was very close in position and height to the lowest migration value in the histogram (Fig. 4A). While it was theoretically possible that a nonzero peak might be found with a finer resolution, these estimates were considered effectively zero (see also Won & Hey, 2005; Dixon, Kapralov & Filatov, 2011). The mean time estimate with statistical confidence (95% highest posterior density) for *t*_1_ (G1:G2) was 1.610 (0.908–2.768), while *t*_2_ (G1:G3) was 1.354 (0.568–3.328). When applying a mutation rate of 12% myr^−1^, the real time *T*_1_ amounted to 21.4 (12.1–36.8) and *T*_2_ was 18.0 (7.5–44.2) kyr (Fig. 4B), roughly corresponding to the LGM happening about 19–22 kyr ago (Yokoyama et al., 2001).

### Microsatellite-based population structure

For microsatellites, genetic diversity indices for each population were summarized in Table 1. The mean allele richness varied from 14.27 in PT to 17.63 in PL, and the observed and expected heterozygosities ranged from 0.81 to 0.90 and from 0.48 to 0.67, respectively. All pairwise *F*_ST_ tests except one were significant after Bonferroni corrections (Table 3). Extremely high *F*_ST_ values (*θ* = 0.056–0.085) were observed between PT and all other populations, suggesting significant genetic differentiation between them.

**Figure 5:**
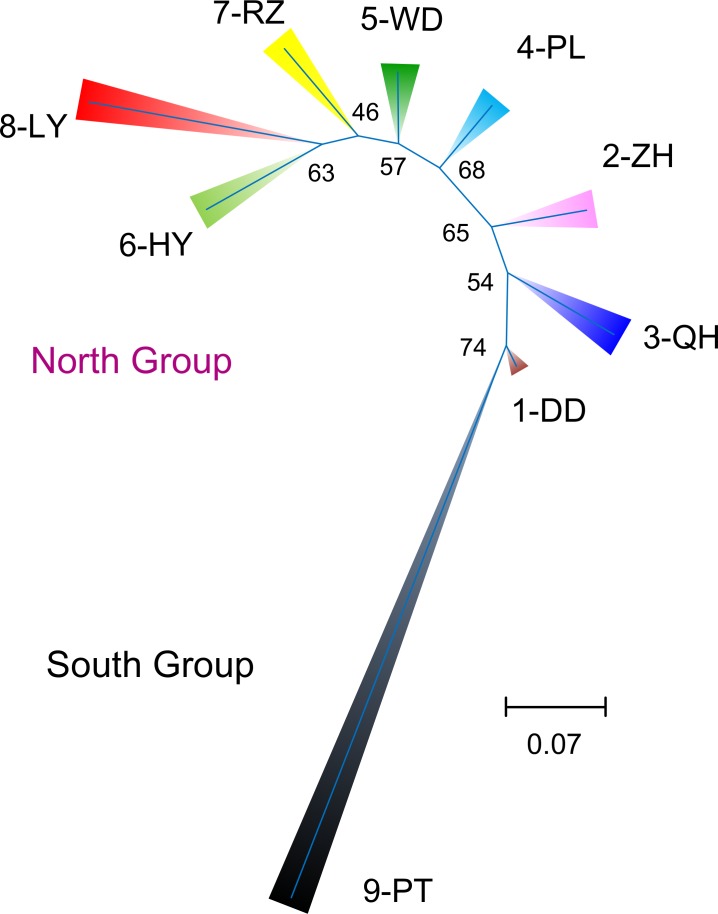
Neighbor-joining tree using microsatellites. Unrooted neighbor-joining tree based on microsatellite *D_C_* distances among the nine populations. Numbers on braches indicate bootstrap support values.

**Figure 6:**
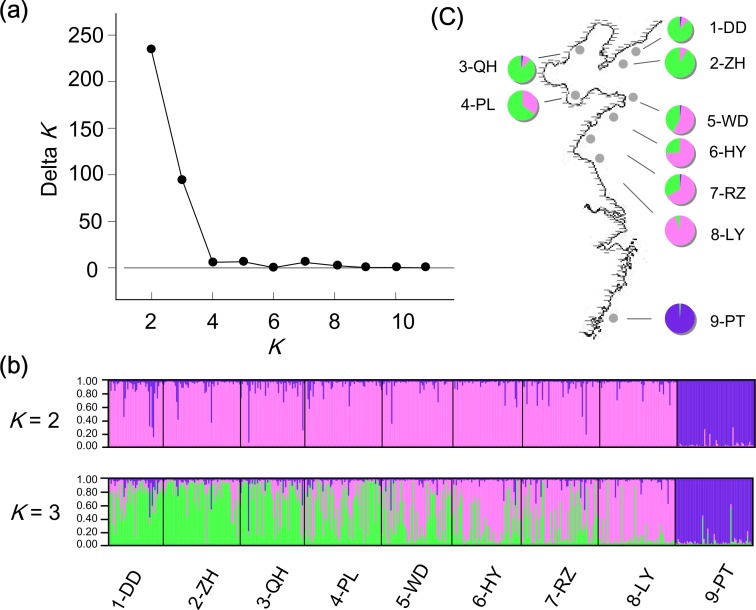
STRUCTURE analyses. Microsatellite-based population structure analyses in STRUCTURE for *M. chinensis*. (A) Estimating the true number of clusters with Δ*K*. Here the uppermost level appears at *K* = 2; (B) the population structure of *M. chinensis* at *K* = 2, 3 respectively; (C) when *K* = 3, the percentage of individuals allocated to each cluster with membership >80% in each population, showing latitude gradations in allele frequencies.

Significant positive correlation was detected between genetic and geographic distance matrices for the nine populations (*R*^2^ = 0.705, *P* = 0.001; Fig. 3B). Population structure revealed by two different analyses displayed a similar pattern as two genetically different groups were observed. According to the NJ tree, the populations were divided into two groups: one consisting of the southern PT, and the other including all other populations northern of the estuary (Fig. 5). For the STRUCTURE analyses, the highest Δ*K* in Structure Harvester was found for *K* = 2 (Fig. 6A). At this value, the result indicated PT dominated by the purple cluster was significantly differentiated from all other sites (Fig. 6B). At *K* = 3, although no additional population group was detected, two clusters (namely pink and green clusters) were recovered with clear gradations in allele frequencies across the northern populations (Figs. 6B and 6C).

## Discussion

In this study, a star-like haplotype topology with shallow divergence was recovered for COI haplotypes of the clam *M. chinensis*, suggesting the single origin of current populations from a common one. Ancestral individuals might have retreated into the ECS refugium when sea levels declined during periods of glaciations, forming a panmictic population. When sea levels rose as glaciers melted after the LGM, the survived individuals migrated out the basin (refugium) and repopulated along the ECS coastline quickly. This might represent a common evolutionary scenario for marine species inhabiting the ECS, and similar patterns were revealed in diverse organisms including fishes, molluscs and crustaceans (Ni et al., 2014). A caveat in this study was that the mitochondrial data (14–23 sequences) for each population had the potential of missing some low frequency haplotypes. Although no divergent lineages were inferred for *M. chinensis*, substantial population subdivision was detected among populations using both COI and microsatellite markers, with somewhat different results: three groups of populations were defined in SAMOVA analysis of mitochondrial COI and a significant level of genetic structure was revealed among them with 65.7% of the total variation explained. Based on “time dependency molecular rates” hypothesis, coalescence analyses suggested the divergence between two pair groups happened about 21.4 and 18.0 kyr ago, respectively, with a close link to the LGM; two groups were supported by microsatellite analyses and the AMOVA result identified a significant among-group component, explaining 5.21% of the total variance (Table 2).

### Population subdivision across the Changjiang freshwater boundary

The most striking result was the significant divergence of PT in the south of Changjiang estuary from the northern ones, congruent with the ‘biogeography and phylogeography concordance’ hypothesis. The mitochondrial data showed that no haplotypes were shared between them and significant differentiation was revealed in pairwise Φ_ST_ analyses. The microsatellite results also supported their division in both *F*_ST_ and population structure analyses (the NJ tree and STRUCTURE). This sharp genetic discontinuity across the estuary most likely results from the influence of the biogeographic boundary associated with habitat discontinuity: several ocean currents and the Changjiang freshwater outflow meet around the estuary, causing striking physical and ecological gradients that may potentially limit gene flow (Su & Yuan, 2005); additionally, the PT site is geographically distant from other populations with the shortest straight-line distance >1,000 km, and there lacks stepping-stone populations (Xu, 1997), which may amplify the barrier effect. The similar effect was also reported for other sympatric species, such as gastropod *Cellana toreuma* (Dong et al., 2012) and two varieties of Sargassum (Cheang, Chu & Ang, 2008). However, it is not consistent across all species as no genetic break linked to the freshwater boundary was noticed for bivalves *Atrina pectinata* (Liu et al., 2011) and *Cyclina sinensis* (Ni et al., 2012). Difference in life-history characteristics, habitat requirement and historical distribution may be responsible for the discordance (Ni et al., 2012).

### Population substructure of the northern group

Besides the divergence of PT, population subdivision was also observed among sites on the same side of the estuary. For the northern populations, the mitochondrial data revealed remarkable differentiation between LY and the rest of the populations both in phylogenetic and pairwise Φ_ST_ analyses, and all of its haplotypes were private. It was an interesting pattern as LY was only ∼50 km apart from the nearest population RZ, without known barriers or steep environmental gradients in such a short distance (Su & Yuan, 2005). IMa analysis suggested effectively zero migration between LY and all other northern populations. The result suggests that PLD periods and ocean currents alone may not be an entirely accurate estimator of realized dispersal. Although the relationship between dispersal potential and gene flow is still a matter of intense debate (e.g., Taylor & Hellberg, 2003; Hellberg, 2009), a comprehensive review by Weersing & Toonen (2009) suggested average PLD of 300 species was only responsible for <10% of variance in measures of gene flow. Additionally, many clams with a sedentary life history usually occur in sheltered nearshore regions to facilitate larval settlement (Wang & Wang, 2008), potentially impeding the exposure of larvae to the open sea and being transported far away (Zhao et al., 2007). A significant IBD pattern revealed in both data sets was coincident with the limited gene flow. Fine-scale genetic structure of molluscan populations was also reported for other molluscs such as abalone *Haliotis diversicolor* (Jiang, Wu & Huang, 1995) and mussels *Mytilus* (Gilg & Hilbish, 2003).

Although no northern population was separated in STRUCTURE analyses of microsatellites, two genetic clusters with clear latitudinal clines were discovered, probably reflecting the influence of environmental gradient factors (e.g., the temperature). Populations on both ends may be more likely to diverge from other ones. For example, LY was found with distinct genetic composition as majority of the individuals (81.7%) could be allocated to the pink cluster with membership >80% (Fig. 6C), compared with the second large proportion of 48.1% in HY (Yates’ chi-squared test: *χ*^2^ = 12.7369, *P* = 0.00036, corrected for continuity).

## Conclusions

Using the surf clam *M. chinensis* as a case study, our results indicate that, although derived from a single ancestral population, significant subdivision can arise for intertidal species within the ECS. Genetic discontinuity was observed between distant populations across the Changjiang Estuary but also between adjacent northern populations, reflecting the influence of multiple driving forces including the Changjiang freshwater boundary, habitat discontinuity, environmental gradient factors and life-history traits on shaping the phylogeographic pattern of *M. chinensis*. Although phylogeographic studies on the NW Pacific have been increasing in recent years, the emerging picture is still far from complete, especially regarding the detailed within-sea evolutionary process (Ni et al., 2014). This study complemented our understanding of possible origins of marine biodiversity in the northwestern Pacific, and highlight the contribution of multiple ecological factors during the process.

## Supplemental Information

10.7717/peerj.1240/supp-1 Supplemental Information 1 Microsatellite raw data

10.7717/peerj.1240/supp-2 Supplemental Information 2 57 COI haplotypes

10.7717/peerj.1240/supp-3 Supplemental Information 3 All COI sequences

## Additional Information and Declarations

### DNA Deposition

The following information was supplied regarding the deposition of DNA sequences: DNA sequences were deposited in GenBank, accessions numbers KC205870–C205926; Microsatellite loci were deposited in GenBank, accession number for Mc1: GQ499856; Mc2: GQ499857; Mc9: GQ499861; Mc10: GQ499862; Mc16: GQ499864; Mc21: GQ499866; Mc24: GQ499868; Mc28: GQ499872; Mc30: GQ499873.
